# Establishment of Local Diagnostic Reference Levels for Adult CT Brain in Johannesburg, South Africa: A Retrospective Protocol Study

**DOI:** 10.3390/diagnostics16091404

**Published:** 2026-05-06

**Authors:** Khahliso Genious Seekoei, Nape Matheko Phahlamohlaka, Jeanette Du Plessis, Setlhapelo Edward Mokhure

**Affiliations:** 1 Department of Clinical Sciences, Central University of Technology, Free State, Bloemfontein 9300, South Africa; nphahlamohlaka@cut.ac.za (N.M.P.); duplesj@cut.ac.za (J.D.P.); 2 Radiation Oncology Department, Charlotte Maxeke Johannesburg Academic Hospital, Park Town, Johannesburg 2193, South Africa; mokhure@yahoo.com; 3 Department of Medical Physics, University of Witwatersrand, Johannesburg 2017, South Africa

**Keywords:** computed tomography, diagnostic reference levels, contrast-enhanced brain, non-contrast-enhanced brain, dose–length product, volumetric CT dose index, effective dose

## Abstract

**Introduction**: Computed Tomography (CT) brain imaging provides high-resolution anatomical detail but involves relatively higher radiation doses, necessitating dose monitoring and optimisation. Diagnostic reference levels (DRLs) are recommended dose indicators for optimising radiation exposure without compromising diagnostic image quality; however, national DRLs for CT brain imaging have not yet been established in South Africa. This article presents a protocol for establishing local DRLs for non-contrast- (non-CE) and contrast-enhanced (CE) adult CT brain examinations at an academic hospital in Johannesburg, South Africa. **Materials and Methods**: The research site is at a single hospital in Johannesburg, South Africa. The research design for this study is retrospective. A sample of 197 adult CT brain examinations (63 non-CE, 34 CE, and 100 combined non-CE and CE examinations) performed between 1 January and 31 December 2024 will be used to develop local DRLs. The 64-slice CT scanner of choice for data collection is the Siemens SOMATOM Definition AS. The population defined for this study is individuals aged 18–70 years. The preferred contrast media used for CT brain examination at the research site is 40 mL of Omnipaque 350. The scan range for CT brain is from the base of the skull (foramen magnum) to the vertex, ensuring full coverage of intracranial structures. Dose metrics, including the volumetric CT dose index (CTDIvol) and dose–length product (DLP), will be extracted from archived dose reports. Local DRLs will be established as the 75th percentile values of CTDIvol and DLP for each protocol group. Descriptive statistics (mean, median, and interquartile range) will be used to summarise the data demographics. The effective dose will be estimated by applying a head-specific conversion coefficient to the DLP values. **Results**: As this is a study protocol, results are not yet available. Local DRLs will be reported as the mean, median, and 75th percentile values of the DLP and CTDIvol for non-CE, CE, and for both non-CE and CE CT brain examinations. The effective dose will be estimated by applying a head-specific dose conversion coefficient (k-factor) to the mean DLP values. **Expected Outcomes**: This study is expected to establish local DRLs for adult CT brain examinations, providing baseline data for dose optimisation and supporting the future development of national DRLs in South Africa. **Conclusions**: Establishing local DRLs will support the optimisation of the radiation dose in CT brain imaging to keep the dose as low as reasonably achievable. The DRLs developed for this study will contribute to national and international efforts toward optimising radiation dose during diagnostic X-ray imaging investigations.

## 1. Introduction

Computed tomography (CT) is commonly used to assess brain pathology and trauma and is performed using multidetector CT (MDCT) systems, such as 16-slice, 64-slice, and 128-slice scanners. However, CT brain imaging exposes patients to relatively higher radiation doses than general radiography of the skull because multiple slices are acquired. A routine CT brain examination typically delivers a radiation dose in the range of 20–40 mGy [1,2]. Omer et al. (2020) reported that during CT brain examinations, the mean organ dose equivalent was 91.41 mSv for the lens and 30.47 mSv for the thyroid gland [3]. A whole-brain CT examination that included the orbit, both axial and helical scans across different CT scanners, showed CTDIvol values ranging from 50.9 to 113.3 mGy, while the corresponding lens doses ranged from 42.6 to 103.5 mGy [4]. Although these dose metrics are diagnostically justified, they may vary considerably across institutions, warranting the establishment of diagnostic reference levels (DRLs) for dose optimisation. Diagnostic reference levels are tools that can be used as a dose indicator benchmark to identify unusually high or low doses for X-ray examinations, prompting optimisation of patient imaging practices [5,6].

A research study of adult CT DRLs reported up to a two- to three-fold variation in DRL values for the same CT examinations between different studies, reflecting differences in scanner technology, scanning protocols, dose indices used, and study design across centres [7]. Furthermore, studies indicate that DRLs are not yet established for all X-ray imaging modalities in every country, including CT, with national and local DRLs still lacking in many regions and imaging types [6,7]. The current study supports and responds to an international call from international radiation regulatory bodies such as the International Commission on Radiological Protection (ICRP), promoting the development of DRLs across different diagnostic imaging modalities in different countries for optimisation of radiation dose and image quality.

CT dose metrics, namely the volume computed tomography dose index (CTDIvol) and dose–length product (DLP), together with patient characteristics (age and gender) and scanner-specific factors, are central to the methodology of establishing DRLs for CT imaging [1,8]. The CTDIvol is a parameter that represents the average dose within the scanned volume and reflects scanner radiation output rather than patient dose. It quantifies the radiation exposure to a single slice of tissue from a single rotation of the CT scanner, expressed in milligray (mGy). The dose–length product represents the total radiation dose delivered over the scanned length (DLP = CTDIvol × scan length) [9]. The 75th percentile of CTDIvol and DLP is used to set up DRLs [8,10,11]. Diagnostic reference levels for CT brain examinations are typically reported separately for contrast-enhanced (CE) and non-contrast (non-CE) protocols because contrast administration influences scan parameters, radiation output, and clinical objectives, often resulting in different dose distributions and optimisation requirements for each technique [12,13]. Iodinated contrast agents such as iohexol, iopamidol, and iodixanol are commonly used to enhance vascular structures and improve the detection of brain pathology. A single hospital located in Gauteng province in South Africa, identified for this study, uses iohexol (Omnipaque 350) with a standard volume of 40 mL for CE CT brain examinations.

In South Africa, national DRLs and standardised dose optimisation protocols for CT brain imaging are not available [14,15], raising concerns about how patient radiation doses during CT brain examinations are monitored and tracked. This practical gap in dose optimisation during CT brain examinations warrants the rationale for this study. In the absence of reliable and established DRLs for CT brain in South Africa, variations in radiation doses for CT brain examinations may go unrecognised, limiting opportunities for dose optimisation. Therefore, the establishment and use of DRLs are key to promoting dose optimisation standards and ensuring that radiation doses are kept as low as reasonably achievable (ALARA).

All health care professionals involved in providing medical imaging services to patients have an obligation to uphold the ALARA principle during all X-ray examinations by employing all possible strategies to optimise the radiation dose and minimise potential health risks associated with radiation exposure to patients. Therefore, the significance of this study lies in the establishment of DRLs to guide radiographers in optimising the radiation dose during CT brain examinations. The results of the current study will help address the limited data on DRLs for non-CE and contrast CE CT brain examinations in South Africa. Thus, the relevance of the research outcome is expected to extend beyond the local context and have a broader impact. To this end, this study will also propose a monitoring tool to aid the implementation and ongoing use of DRLs in adult CT brain imaging at the selected hospital, to keep radiation exposure to patients as low as reasonably achievable.

## 2. Aim and Objectives

The study aims to establish local DRLs for non-CE and CE adult CT brain examinations at a single academic hospital in Johannesburg, Gauteng province. The objectives of this study are:

- Calculate the mean, median, and 75th percentile of CTDIvol and DLP.
- Estimate effective doses of non-CE and CE CT brain examinations.
- Compare the established local DRLs from this study with internationally published DRLs for adult patients with CT brain.
- Propose a DRL monitoring tool to guide the implementation of DRLs for adult CT brain imaging at the selected academic hospital

## 3. Materials and Methods

### 3.1. Study Design

This study will adopt a retrospective research design. CT dose metrics for brain examinations will be extracted from archived dose reports available in the Picture Archiving and Communication System (PACS). The research setting for this study will be a single academic hospital in the Gauteng Province, South Africa.

The research setting for this study will be at one of the large academic hospitals in Gauteng Province, South Africa. To support the accuracy and reliability of the data to be collected, a quality control test will be performed on the CT scanner to verify its X-ray output, thereby justifying the experimental component of the study.

### 3.2. Population and Sampling

The study population will comprise datasets of adult patients who underwent CT brain examinations at the selected academic hospital between 1 January 2024 and 31 December 2024. The study will include standard adult patients aged between 18 and 70 years. A convenience (non-probability) sampling strategy will be used to select datasets eligible for CT brain examinations within the study period. This approach is appropriate for DRL studies, where the objective is to obtain a large, representative dataset available and accessible on the PACS to develop local DRLs [16]. A target sample size of 197 brain CT examinations will be used, including non-CE (63 brain scans), CE (34 brain scans), and both datasets of patients who underwent both non-CE and CE (100 brain scans). Table 1 presents the inclusion and exclusion criteria for data selection.

## 4. Data Acquisition

**Figure 1:**
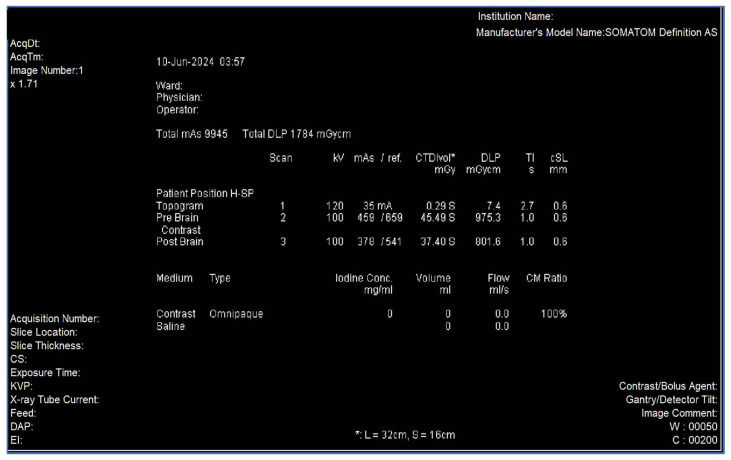
An example of a dose report for non-CE and CE CT brain.

Data will be retrospectively extracted from archived PACS dose reports. The CT brain scans identified for this study would have been acquired using a 64-slice Siemens (Berlin, Germany) SOMATOM Definition AS scanner (syngo CT VA48A). Dose reports will serve as the primary data source for extracting dose indices, including the CTDIvol and DLP (see Figure 1). Accessible data on CT scan parameters, such as kVp, mAs, slice thickness, and scan length, will also be captured. Table 2 provides an overview of the routine adult CT brain protocol scanning parameters. Patient demographics, including age and gender, will be obtained from the radiology information system. A Microsoft Excel spreadsheet will be used to capture and manage research data.

### 4.1. Data Reliability and Validity

**Figure 2:**
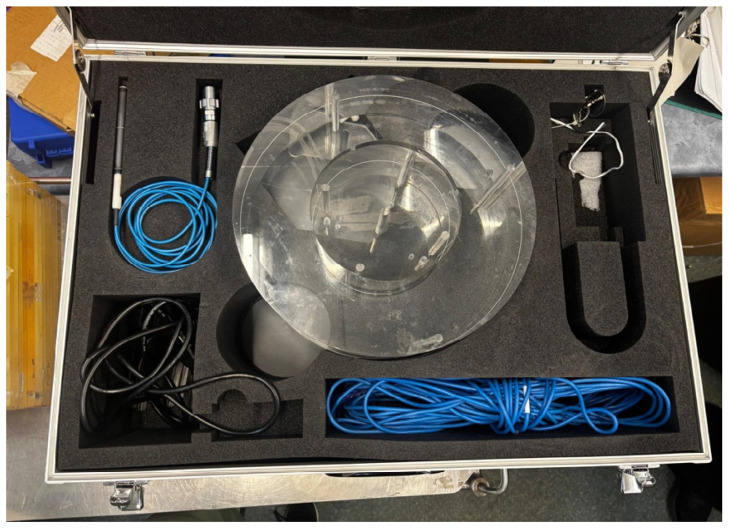
A 16 cm head acrylic phantom.

**Figure 3:**
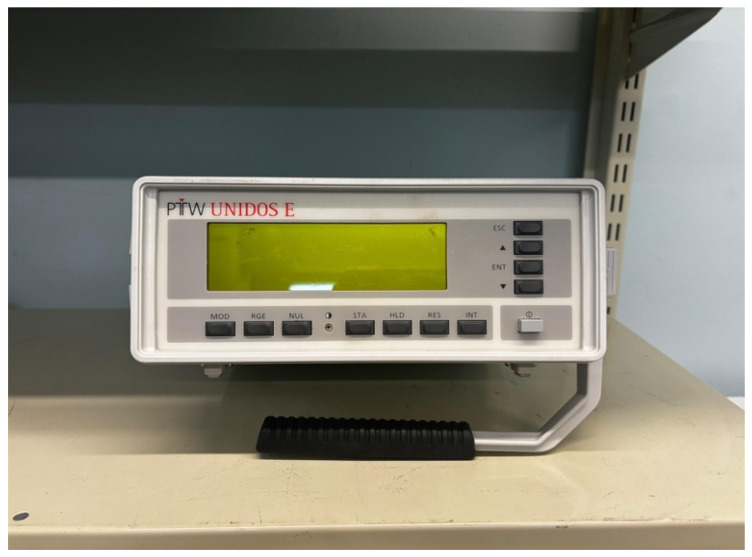
An electrometer to measure the dose output of a CT scanner.

Before CT data extraction on the PACS, an audit of the CT scanner’s individual equipment record file, including verification of the machine’s licence, will be conducted. This audit will confirm whether all required quality control tests were performed during 2024 in accordance with the South African Health Products Regulatory Authority (SAHPRA) guidelines [19] and whether appropriate, validated instrumentation was used (e.g., the correct phantom and electrometer), as shown in Figure 2 and Figure 3. Records will also be reviewed to confirm that the electrometer calibration was performed in accordance with the calibration standards of the National Metrology Institute of South Africa (NMISA). Finally, the CT scanner’s X-ray output measurements will be verified to ensure that they are within the acceptable tolerance limits of the established baseline values [20].

### 4.2. Statistical Analysis

Descriptive statistics (mean, median, and standard deviation) will be computed to summarise the CT and patient demographic data. The interquartile range (IQR) will be calculated for the CTDIvol and DLP to characterise dose variability using a Microsoft Excel spreadsheet and verified using IBM SPSS Statistics software version 30.

### 4.3. Diagnostic Reference Levels Calculations

The establishment of DRLs will be conducted in line with internationally accepted guidelines for radiation protection and dose optimisation in medical imaging [21]. The DRL value will be calculated as the third quartile (75th percentile) of the dose value using Equation (1) in a Microsoft Excel sheet. To find the third quartile (Q3) position from the distribution of patient dose data, the Q3 value will be calculated using Equation (2) [22].

(1) $$n_{q3}=\frac{3(n+1)}{4}$$

In addition, the Q3 value was obtained from Equation (2).

(2) xq3=xa3+14(xb,3−xa,3)

The 75th percentile (third quartile) will be computed for each parameter using the following formula:

(3) $$P_{k}=\frac{k}{100}\times (n+1)$$

where k = 75 and n is the total number of observations.

DRLs will be reported as the 75th percentile for the CTDIvol and DLP values within each protocol group. The calculated DRLs will be compared with the published international DRLs to assess compliance and identify optimisation opportunities.

DRL = P_k_ (Dose distribution) (4)

where P_k_ is the 75th percentile.

### 4.4. Estimation of Effective Dose

The effective dose will be estimated using a region-specific conversion coefficient (k-factor = 0.0021 mSv/mGy·cm) for adult head CT, based on published guidelines from the American Association of Physicists in Medicine (AAPM) and ICRP recommendations [22].

### 4.5. An Overview of the Process for Establishing Local Diagnostic Reference Level

**Figure 4:**
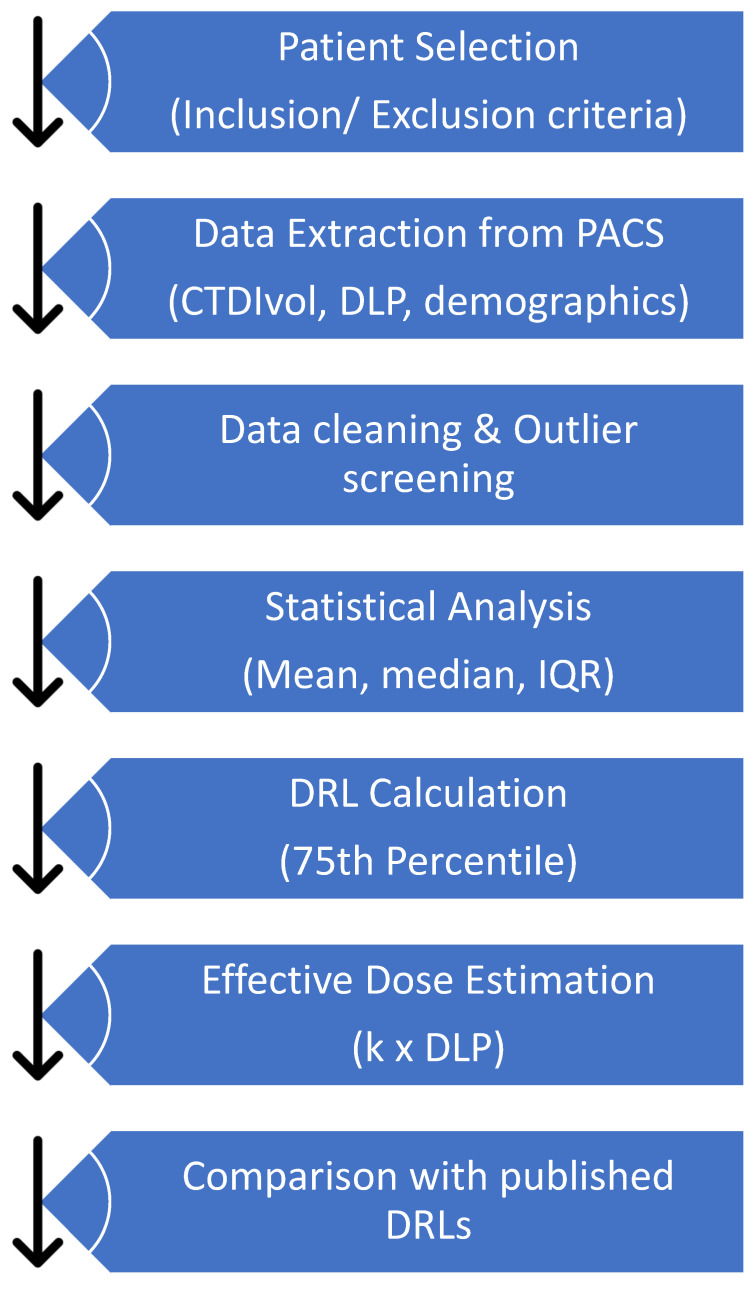
A process to guide the development of a diagnostic reference level.

The flowchart in Figure 4 presents a methodological approach for developing local DRLs for non-CE and CE brain examinations in the current study. First, brain scans of eligible patients will be retrieved from the PACS. Secondly, relevant dose metrics, including the CTDIvol, DLP, and demographic data, will be extracted from the PACS. After that, the data will undergo cleaning and analysis using statistical measures such as the mean, median, and IQR. The DRLs in this study will be established at the 75th percentile, and the effective dose is estimated using the formula k × DLP. Finally, the results will be compared with published DRLs to inform protocol optimisation and facilitate continuous monitoring of radiation exposure in patients undergoing CT brain examinations.

### 4.6. Ethical Aspects and Data Management

The research protocol was approved by the Faculty Research and Innovation Committee (FRIC) for the Master of Radiography at the Central University of Technology (CUT), Free State. The study is registered with the National Health Research Database (NHRD) (Ref: GP_202502_069). Gatekeeper approval was obtained from the deputy manager of the X-ray departments at the participating hospital and the Gauteng Department of Health. Ethical approval was granted by the University of the Witwatersrand Health Research Ethics Committee (Ref: M250449). As the study uses retrospective data without direct patient involvement, informed consent was not required. All data will be anonymised and securely stored on a password-protected laptop and the CUT SharePoint drive, accessible only to the researcher, supervisors, and biostatistician, in compliance with the Protection of Personal Information Act (POPIA).

## 5. Discussion

The outcomes of this research will contribute to the expanding body of knowledge on DRLs for optimising CT brain imaging, with a particular emphasis on image quality and radiation dose in the context of South Africa. The DRLs in this study will be calculated as the 75th percentile CTDIvol and DLP and reported along with the effective doses for CT brain, including non-CE, CE, and for both non-CE and CE examinations. In Poland, the 75th percentile DRLs for both non-CE and CE head CT were 58.18 mGy (CTDIvol) and 1018.11 mGy·cm (DLP) per acquisition, with total DLPs of 2046.09 mGy·cm for CE and 1027.99 mGy·cm for non-CE scans [13]. Slightly lower but comparable values were reported in a Korean national survey for adult non-CE brain CT (52.2 mGy and 969.8 mGy·cm), suggesting effective dose optimisation [23]. In contrast, higher DRLs were observed in Northern Jordan (76 mGy and 1388.5 mGy·cm), indicating regional variation and potential for further optimisation [24]. Notable differences are observed between countries, highlighting the influence of scanner settings, optimisation practices, and local dose management policies. These results underscore the importance of establishing both national and local DRLs to guide dose optimisation for specific CT brain indications.

Table 3 summarises published DRLs for CE and non-CE brain CT examinations across different countries. The CTDIvol and DLP values vary considerably depending on the clinical indication, contrast use, and regional protocols, with CE scans generally showing higher dose values than non-CE scans.

In this study, the effective dose will be calculated from mean DLP values to enable population-level dose comparisons rather than to assess individual patient exposure. For non-CE and CE brain CT examinations, the effective dose will be estimated using a head-specific DLP conversion factor, facilitating protocol comparison and radiation dose optimisation rather than individual risk evaluation. It is important to emphasise that the effective dose is derived from the DLP using region-specific conversion factors for adult head CT based on ICRP recommendations. These conversion coefficients were developed using Monte Carlo simulations of standard phantoms and ICRP tissue weighting factors and are most useful for protocol comparison and optimisation across populations, rather than individual patient risk quantification [9,29].

Based on ICRP Publication 103 [30] and Monte Carlo simulations of standard phantoms, a commonly used DLP-to-effective dose conversion factor for adult head CT is approximately 2.1 µSv per mGy·cm (0.0021 mSv/mGy·cm) for head CT examinations. Other clinical series and dosimetry surveys align with these values, with typical quoted values around 1.5 mSv for a non-contrast head CT in adults [31,32].

From the results of this study, a protocol will be proposed to educate and guide every health professional involved in the imaging of patients on how to implement DRLs for adult CT brain imaging in practice at the selected hospital. This protocol will serve as a practical tool to support a bid to use dose optimisation strategies during CT brain imaging through DRLs. This study is important as it intends to fill this gap by developing local DRLs for adult CT brain imaging and comparing them to internationally published DRL values, thereby contributing to global discussions on dose optimisation during CT imaging.

## 6. Limitations of the Study

The retrospective nature of this study design limits access to patient weight data and clinical indications to establish weight-based and clinically based DRLs. The adopted research design also does not consider the variability of routine CT brain scanning protocols, as scanning techniques and parameter selection may vary between operators or evolve. The research data will be collected from a single CT scanner at one hospital in one city. Although this ensures data uniformity, it cannot reflect dose variations between scanner models or technologies and the patient population, limiting the generalizability of the results. Lastly, this study will not assess the correlation between image quality and dose.

## 7. Conclusions

This research study envisages to establish local DRLs for adult CT brain scans at a single hospital in Johannesburg, thereby contributing to the emerging data on DRLs in South Africa. Additionally, the results of this study will offer institutionalised DRLs that can serve as a benchmark for comparison and aid the development of national DRLs in the South African context. Future research should integrate data from multiple centres, encompassing patient demographics, clinical indications, and weight metrics. Finally, other researchers should also examine the correlation between the radiation dose and image quality to further enhance dose optimisation practices for CT brain imaging protocols.

## Figures and Tables

**Table 1:** Inclusion and exclusion criteria.

Inclusion	Exclusion
Non-CE and CE CT brain examinations	Arterial CT brain/neck examinations
CT brain examinations performed between 1 January 2024 and 31 December 2024	Brain CT examinations performed before 1 January 2024
CT brain scans of patients between 18 and 70 years old	CT brain scans of patients aged <18 years

**Table 2:** Routine adult CT brain protocol parameters [17,18,19].

Parameter	Value
Scanner	Siemens SOMATOM Definition AS
Tube Voltage (kVp)	100 to 120
Tube Current (mAs)	100 to 659
Slice Thickness (mm)	Range 1 to 5
Pitch	Range 0.5 to 2
Rotation Time (s)	1.0
Scan Length (mm)	110 to 121
Contrast Agent (CE only) (mL)	Iohexol (Omnipaque 350/300), 40
Collimation (mm)	128 × 0.6

**Table 3:** Comparison of DRLs for CE and non-CE CT brain examinations.

Country	Indication	CTDIvol (mGy)	DLP (mGy·cm)	NDRL/LDRL
Smith-Bindman et al. [25] (Germany)	Contrast-enhanced brain (local)	58.2	1018	NDRLs
Albadarneh et al. Hayton et al. [24,26] (Australia)	Non-contrast brain	52	880	NDRLs
Paulo et al. [27] (UK)	Acute Stroke	80	—	NDRLs
Modlińska et al. Paulo et al. [13,27] Switzerland/Denmark	Post-contrast brain (trauma)	65	1000	NDRLs/LDRLs
Modlińska et al. [13] Austria	Post-contrast brain (sinusitis)	—	90	LDRLs
Paulo et al. [27] France	Post-contrast brain (trauma)	44	1010	NDRLs
Uushona et al. [28] Ghana, Kenya, Namibia and Senegal	Non-contrast brain	60.9	1259	NDRLs

## Data Availability

Not applicable.
